# Gut microbiota comparison of vaginally and cesarean born infants exclusively breastfed by mothers secreting α1–2 fucosylated oligosaccharides in breast milk

**DOI:** 10.1371/journal.pone.0246839

**Published:** 2021-02-08

**Authors:** Karina M. Tonon, Tania B. Morais, Carla R. Taddei, Humberto B. Araújo-Filho, Ana Cristina F. V. Abrão, Antonio Miranda, Mauro B. de Morais

**Affiliations:** 1 Nutrition Postgraduate Program, Universidade Federal de São Paulo, São Paulo, Brazil; 2 Food Quality Control Laboratory, Universidade Federal de São Paulo, São Paulo, Brazil; 3 Department of Clinical and Toxicological Analysis, Universidade de São Paulo, São Paulo, Brazil; 4 School of Arts, Sciences and Humanities, Universidade de São Paulo, São Paulo, Brazil; 5 Division of Pediatric Gastroenterology, Universidade Federal de São Paulo, São Paulo, Brazil; 6 Breastfeeding Incentive and Support Center, Universidade Federal de São Paulo, São Paulo, Brazil; 7 Department of Biophysics, Universidade Federal de São Paulo, São Paulo, Brazil; University of Minnesota Twin Cities, UNITED STATES

## Abstract

**Background:**

Exclusive breastfeeding promotes beneficial modifications on the microbiota of cesarean born infants, but little is known about the role of specific breast milk components in this modulation. Women with an active FUT2 gene (called *secretors*) secrete α1–2 fucosylated human milk oligosaccharides (HMOs), which promote *Bifidobacterium* in the infant’s gut and may modulate the microbiota of cesarean born infants.

**Objective:**

To compare the microbiota composition of cesarean and vaginally born infants breastfed by secretor mothers.

**Methods:**

Maternal secretor status was determined by the occurrence of 4 different α1–2 fucosylated HMOs in breast milk by LC-MS. The fecal microbiota composition from cesarean and vaginally born infants was analyzed by 16S rRNA gene sequencing and qPCR, stratified by the maternal secretor status, and compared.

**Results:**

Alpha and beta diversity were not significantly different in cesarean born, secretor-fed infants (CSe+) compared to vaginally born, secretor-fed infants (VSe+). There were no significant differences in the fecal relative abundance of *Bifidobacterium* between CSe+ and VSe+ infants, but the prevalence of the species *B*. *longum* was lower in CSe+. The fecal relative abundance of *Bacteroides* was also lower, while *Akkermansia* and *Kluyvera* were higher in CSe+ infants.

**Conclusion:**

Cesarean and vaginally born infants fed with breast milk containing the α1–2 fucosylated HMOs fraction present similar amounts of *Bifidobacterium* in the feces, but differences are observed in other members of the microbiota.

## Introduction

Disruptions in the natural assembly of the neonatal microbiota have been associated with increased risk of immune-mediated diseases [1,2] and excessive weight later in childhood [3–5]. The mode of birth is one of the major factors that significantly affect bacterial colonization and the development of the gut microbial community [6]. Vaginally born infants harbor a microbiota enriched with mutualistic bacteria such as *Bifidobacterium*, *Escherichia*, *Bacteroides*, and *Parabacteroides*. In contrast, cesarean born infants are depleted of these genera and are instead dominated by *Enterococcus*, *Staphylococcus*, *Streptococcus*, *Klebsiella*, *Enterobacter*, and *Clostridium*, which are commonly associated with the human skin and the hospital environment [6–10].

Previous studies demonstrated that exclusive breastfeeding can partially restore the disruptions caused by cesarean birth in the infant gut microbiota, without exploring the role of breast milk composition [11,12]. Liu *et al*. reported that cesarean born/exclusively breastfed infants share a more similar gut microbiota with vaginally born/exclusively breastfed than cesarean born/mixed-fed infants [12]. Recently, a study showed that 2’-fucosyllactose (2’FL) as an indicator of maternal secretor status, might be involved in the restoration promoted by exclusive breastfeeding on the gut microbiota of cesarean born infants [13]. So far, this is the only evidence on the role of a human milk component in repairing the changes caused by cesarean section on the infant gut microbiota.

Human milk oligosaccharides (HMOs) comprise the third largest solid fraction of human milk, after lactose and lipids, containing more than 150 different molecules with a total concentration between 5 and 20 g/L in mature human milk [14–16]. HMOs are not digested by the infant and reach the colon intact, where they act as prebiotics, antimicrobials, prevent pathogen binding, and promote the gut barrier function [15]. The composition and concentrations of HMOs are highly variable and influenced by several maternal characteristics, especially the secretor status, determined by the activity of the FUT2 gene [17–19]. FUT2 encodes the enzyme fucosyltransferase 2, which adds a fucose residue in an α1–2 linkage to the HMO chain [20,21]. Secretor women have an active FUT2 enzyme and produce high amounts of α1–2 fucosylated HMOs, such as 2’FL and lacto-N-fucopentaose I (LNFP I).

In contrast, due to a single nucleotide polymorphism in FUT2, non-secretors produce no or only small amounts of α1–2 fucosylated HMOs [13]. Breastfed infants from secretor mothers are colonized earlier by *Bifidobacterium* and present higher amounts of this genus in the feces than non-secretor-fed infants [22,23]. Furthermore, isolated *Bifidobacterium* from secretor-fed infants can consume 2’FL and distinct sets of *Bifidobacterium* dominate the microbial community in secretor-fed and non-secretor-fed infants [23].

Therefore, we hypothesized that besides the birth mode, the maternal secretor status (or the presence of α1–2 fucosylated HMOs in breast milk) also influences the fecal microbiota of exclusively breastfed infants. The purpose of this study was to compare the microbiota composition of cesarean and vaginally born infants breastfed by secretor mothers.

## Materials and methods

### Participants and sample collection

The participants of the study were a subset of mother-infant pairs enrolled in a cross-sectional, observational study whose aim was to identify maternal and infant factors associated with HMOs concentrations [19]. Mothers and their infants were included at one month postpartum (median: 34 days, 25th-75th percentile: 25–45 days postpartum). Data and samples were collected on the same day of enrollment in the study. Healthy full-term (gestational age ≥ 37 weeks), singleton, exclusively breastfed infants were included. Infants that received antibiotic treatment, probiotics, water, or any other food besides human milk were not enrolled. The inclusion criteria for the present study were the availability of a human milk sample from the mother and a stool sample from the infant. From the 78 pairs included in the original cohort, 54 provided both samples and were selected for this subset (S1 Fig).

Human milk samples (5 to 15 mL) were obtained by manual expression of the breast during the morning (8:30–12:00 a.m.) and stored at −20°C until HMOs analysis. Infant feces were collected from disposable diapers and transferred to a microfuge tube containing 1 mL of the ASL buffer from the QIAamp DNA Stool Mini Kit (Qiagen, Hilden, Germany) and stored at −20°C until DNA extraction. Human milk and infant feces samples were collected on the same day.

### Ethics approval and consent to participate

This study was approved by the Ethics Committee of the Universidade Federal de São Paulo (protocol No. 419.162) and complied with the Declaration of Helsinki. All mothers received detailed oral and written information about the study and voluntarily agreed to participate. Written informed consent was obtained from each participant before the data and sample collection.

### Maternal secretor status determination

Maternal secretor status was determined based on the presence of α1–2 fucosylated structures in the human milk sample by liquid chromatography-mass spectrometry (LC-MS), as previously described in detail [24]. Briefly, after fat and protein removal, the human milk sample was diluted and subjected to a reduction reaction with 0.25M sodium borohydride. The resulting extract containing the HMOs fraction was injected into the LC-MS system for HMOs analysis. Mothers whose human milk sample presented at least one of the α1–2 fucosylated structures 2’-FL, LNFP I, lacto-N-difucohexaose I (LNDFH I), and difucosyllacto-N-hexaose c (DFLNHc) were ascribed as secretors, and those that did not present any of those HMOs up to quantifiable amounts (0.039 μg/mL for 2’-FL, LNDFH I and DFLNHc and 0.156 μg/mL for LNFP I) were ascribed as non-secretors.

### Fecal microbiota analysis

Whole genomic DNA was extracted from feces using the QIAamp DNA Stool Mini Kit (Qiagen, Hilden, Germany) following the manufacturer’s instructions. Purified DNA was diluted to a final volume of 200 μL, and DNA quantification was performed using a spectrophotometer, model Denovix DS-11 (Denovix, Delaware, USA). All DNA samples were diluted to a final concentration of 20 ng/μL and stored at -20°C.

The overall fecal microbiota composition was analyzed by 16S rRNA gene sequencing. The bacterial 16S rRNA gene, hypervariable region V4, was amplified by PCR using V4 primers with specific Illumina adaptors [25]. Amplification of the target sequences was performed in two stages, following the instructions of the Illumina protocol (Illumina®, California, United States). After amplification, the samples were grouped in equimolar amounts, with a final concentration of 12 pM and sequenced using the 500-cycle Miseq V2 Kit (Illumina®, California, United States), including 20% PhiX as an internal control for low libraries diversity. Besides, all reagents and ultrapure water were used as blank samples, and no signal was detected in the sequencing cartridge, indicating that bacterial contamination was minimal during library preparation and sequencing.

The main bacterial genera and species were quantified by qPCR using specific primers (S2 Table). The qPCR reactions were conducted on the Rotor-gene Q (Qiagen) equipment, in duplicate, in a final volume of 10 μL. The qPCR conditions were as follows: initial denaturation at 95°C for 5 minutes, 40 cycles at 95°C for 10 s, and a final stretching step at 60°C for 15 s. The dissociation cycle of the products for the melting curve was 95°C for 1 minute and a step to perform the denaturation curve with a variation of 70°C to 95°C, with a gradual increase in temperature of 1°C/s.

The standard curve for the quantification was created by TopoTA cloning plasmids (Invitrogen, Carlsbad, CA, USA), containing the reference gene fragment for each bacterium, previously amplified by PCR. With the molecular mass of the plasmid and the size of the insert, it was possible to calculate the number of copies of the genes according to the formula: mass in Daltons (g/mol) = (double-strand size [ds] amplicon in base pairs [bp]) (330 Da × 2 nucleotides [nt] / bp). In this way, dividing the concentration in g/mol by the Avogadro constant, the number of molecules/g was obtained, which is equal to the number of copies/g of the gene. As a negative control, samples containing all reagents were used, except for DNA.

From this information, it was possible to determine the number of copies of each gene, providing a standard for the construction of a reference curve for quantification by qPCR. The results of qPCR were expressed as bacterial units/g of feces (U/g of feces). The detection limit was 1 cell/g for all organisms.

### Bioinformatics

The generated fragments were joined and analyzed using the software QIIME version 1.9.1 [26] with each unique sequence assigned to an operational taxonomic unit (OTU) based on ≥ 97% similarity by the UCLUST algorithm, representing the smallest taxonomic entity and classified phylogenetically using the ribosomal sequences from the SILVA reference database, version 128 [27].

The resulting OTU table was filtered to remove singletons and any OTU with an abundance of less than 0.05% across all samples. The median (25th-75th percentiles) number of sequences found in the samples was 90158 (66398–153979), and the minimum number of sequences in a sample was 7044 sequences. Alpha diversity was analyzed using the number of observed OTUs, Chao1, and Shannon indexes, without rarefaction [28]. Beta diversity was calculated using weighted and unweighted UniFrac distances [29].

### Statistical analysis

Statistical tests were performed to compare the microbiota composition of infants from secretor mothers, stratified in cesarean (CSe+, n = 27) and vaginally born (VSe+, n *=* 21). Non-secretor mothers and their infants were not included in the statistical analysis of the microbiota, but their data were described.

UniFrac distances were associated with birth mode using the method Adonis in QIIME [22]. Analysis of Covariance (ANCOVA) and non-parametric Analysis of Covariance (Ranked ANCOVA or Quade test [30]) were used to verify differences in α-diversity indices, relative abundances of the most abundant phyla and genera, and species absolute concentrations between CSe+ and VSe+ with age at inclusion as a covariate, according to the distribution of each variable. We used a free web program (www.masungur.com) to perform the ranked ANCOVA [31]. Chi-square test or Fisher’s exact test were used to compare the prevalence of bacterial genera and species between the groups. Clinical and demographic characteristics of the study population and the concentrations of α1–2 fucosylated HMOs were described using mean (SD), median (p25 –p75) or proportions and compared using Student’s t-test, Mann-Whitney rank-sum test, Chi-square test, or Fisher’s exact test according to the distribution and nature of the variables. Zero/non-detected levels were included in the comparisons of qPCR data and missing data were reported. Analyses were conducted with the software SigmaPlot version 12.0 (Systat Software, Inc., Chicago, IL) or R version 3.4.4 (The R Foundation for Statistical Computing, Vienna, Austria). The p-values for α-diversity indices, relative abundances of the most abundant phyla and genera, and species absolute concentrations were adjusted for multiple comparisons using the Benjamini and Hochberg method [32] to control the false discovery rate. Results were considered statistically significant when *p* < 0.05.

## Results

Table 1 shows the characteristics of the study population according to the mode of birth and maternal secretor status. No significant differences were observed in clinical and demographic characteristics between the four groups (Table 1).

**Table 1:** Characteristics of the mothers and their exclusively breastfed infants according to the mode of birth and maternal secretor status.

	VSe+ (*n* = 21)	CSe+ (*n* = 27)	VSe- (*n* = 4)	CSe- (*n* = 2)	*p*
Mothers					
Age, years (mean (SD))	31 (6)	31 (7)	31 (6)	26 (2)	0.738^f^
BMI, kg/m^2^ (median (p25 –p75))					
Pre-gestational	24.8 (22.4–27.9)	23.6 (21.5–30.6)	22.5 (21.3–22.7)	22.5 (21.5–23.5)	0.403^g^
At inclusion	26.8 (24.4–29.0)	25.8 (23.3–31.2)	23.3 (22.1–24.8)	25.2 (24.3–26.2)	0.446^g^
Parity, *n* (median (p25 –p75))	2 (1–2)	1 (1–2)	2 (1–2)	1 (1–2)	0.535^g^
Allergic disease, *n* (%)	6 (29)	5 (19)	0 (0)	0 (0)	>0.498^h^
Education, *n* (%) ^a^					
Elementary school	2 (11)	2 (8)	1 (25)	0 (0)	1.000^h^
High school	9 (47)	6 (25)	2 (50)	2 (100)	>0.086^h^
Graduate	5 (26)	11 (46)	1 (25)	0 (0)	>0.221^h^
Postgraduate	1 (5)	5 (21)	0 (0)	0 (0)	>0.205^h^
Infants					
Gestational age at birth, weeks (mean (SD))	38.90 (1.10)	38.97 (1.40)	38.57 (1.41)	38.78 (0.71)	0.960^f^
Birth weight, g (mean (SD))	3097.50 (731.90)	3179.77 (436.62)	3232.50 (202.05)	3097.50 (731.86)	0.922^f^
Age at inclusion, days (mean (SD))	32.4 (10.7)	40.0 (14.7)	28.8 (5.9)	33.5 (19.1)	0.151^f^
Weight at inclusion, g (median (p25 –p75))	4177.50 (2690.00–5665.00)	4250.00 (3677.50–5002.50)	4192.50 (4037.50–4345.00)	4177.50 (2690.00–5665.00)	0.990^g^
Length at inclusion, g (mean (SD))	53.7 (2.4)	54.0 (3.3)	53.5 (1.3)	52.8 (5.3)	0.937^f^
Weight gain, g/day (mean (SD)) ^b^	28.49 (14.82)	26.89 (11.98)	32.35 (8.84)	24.56 (26.95)	0.864^f^
Sex, *n* (%) ^c^					
Male	8 (38)	14 (54)	2 (50)	1 (50)	>0.380^h^
Female	13 (62)	12 (46)	2 (50)	1 (50)	>0.380^h^
Household					
Household with siblings, *n* (%) ^d^	10 (53)	7 (33)	3 (75)	1 (50)	>0.270^h^
Household with pets, *n* (%) ^e^	7 (37)	6 (30)	2 (50)	1 (50)	>0.580^h^

VSe+: Vaginally born, secretor; CSe+: Cesarean born, secretor; VSe-: Vaginally born, non-secretor; CSe-: Cesarean born, non-secretor

^a^ missing data from 2 mothers from group VSe+ and 3 mothers from group CSe+

^b^ Weight gain = (weight at inclusion—birth weight)/age at inclusion

^c^ missing data from 1 infant from group CSe+

^d^ missing data from 2 infants from group VSe+ and 6 infants from group CSe+

^e^ missing data from 2 mother-infant pairs from VSe+ and 7 pairs from group CSe+

^f^ One-way ANOVA

^g^ Kruskal-Wallis test

^h^ Fisher’s Exact Test.

The median (25th-75th percentile) amount of LNFP I was significantly lower in the milk from mothers of cesarean born infants than in the milk from mothers of vaginally born infants (0.511 (0.269–0.962) g/L *vs*. 0.736 (0.444–1.564) g/L, respectively; *p* = 0.025). No significant difference was observed in 2’-FL or the other α1–2 fucosylated HMOs amounts between the groups (S1 Table).

**Fig 1:**
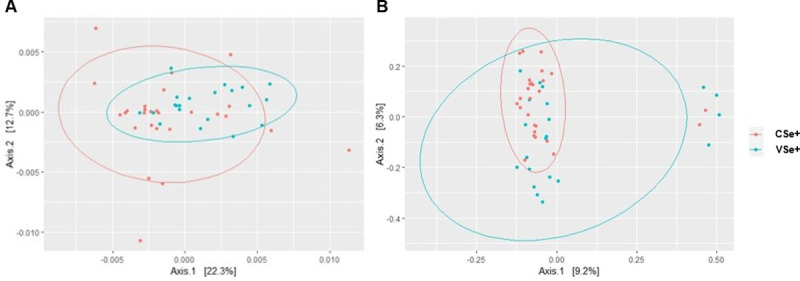
Principal coordinate analysis (PCoA) of the overall microbiota composition of exclusively breastfed infants from secretor mothers by birth mode using (A) unweighted and (B) weighted UniFrac distances. VSe+: vaginal, secretor; CSe+: cesarean, secretor.

The overall microbiota composition did not differ between CSe+ and VSe+ infants when comparing the unweighted (p = 0.75) and weighted (p = 0.87) UniFrac distances using the method Adonis (Fig 1A and 1B). The number of observed OTUs, the richness estimator (Chao1), and the diversity index (Shannon) also did not differ between CSe+ and VSe+ infants, as shown in Table 2.

**Table 2:** Fecal microbiota alpha diversity from vaginally and cesarean born, exclusively breastfed infants from secretor mothers.

Index	VSe+ (*n* = 21)	CSe+ (*n* = 27)	p*
Observed OTUs	595.381 ± 151.699	577.889 ± 214.780	0.841
Chao 1	876.230 ± 169.751	866.394 ± 282.444	0.988
Shannon	1.871 ± 0.412	2.032 ± 0.483	0.394

Values are presented in mean ± SD; VSe+: Vaginally born, secretor; CSe+: Cesarean born, secretor.

*p-values were adjusted for age at inclusion using ANCOVA (analysis of covariance).

**Fig 2:**
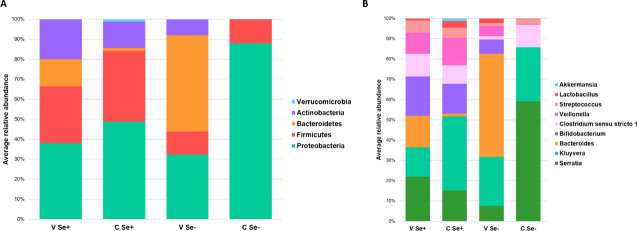
Average relative abundances of the most abundant bacterial phyla (A) and genera (B) of the infant fecal microbiota according to the birth mode and maternal secretor phenotype. The taxa with an average relative abundance < 1% were removed and those with an average relative abundance ≥ 1% were set to 100%; VSe+: Vaginally born, secretor; CSe+: Cesarean born, secretor; VSe-: Vaginally born, non-secretor; CSe-: Cesarean born, non-secretor.

The relative abundance of the most abundant bacterial phyla was not different in CSe+ and VSe+ infants, except for Bacteroidetes and Verrucomicrobia (Table 3, Fig 2A). The relative abundances of the most abundant bacterial phyla and genera of cesarean born, non-secretor fed infants (CSe-, n = 2) and vaginally born, non-secretor fed infants (VSe-, n = 4) are presented in Fig 2 for descriptive purposes and were not included in the statistical analysis.

**Table 3:** The average relative abundance of the most abundant fecal bacterial groups from vaginally and cesarean born, exclusively breastfed infants from secretor mothers.

Group	Average relative abundance (%)		*p*
	VSe+	CSe+	
Phyla			
Actinobacteria	19.75	13.25	0.185^a^
Bacteroidetes	13.81	1.45	0.003*^b^
Firmicutes	28.43	35.36	0.465^a^
Proteobacteria	37.88	48.73	0.094^a^
Verrucomicrobia	0.00	1.08	0.030*^b^
Genera			
*Akkermansia*	0.00	1.18	0.030*^b^
*Bacteroides*	13.94	1.32	0.006*^b^
*Bifidobacterium*	17.43	13.48	0.342^b^
*Clostridium sensu stricto 1*	9.99	8.47	0.149^b^
*Kluyvera*	13.13	33.82	0.001*^b^
*Lactobacillus*	0.87	2.99	0.283^b^
*Serratia*	19.78	13.91	0.950^b^
*Streptococcus*	5.50	4.66	0.975^b^
*Veillonella*	9.38	12.51	0.093^b^

The most abundant taxa were those with an average relative abundance ≥ 1%; VSe+: Vaginally born, secretor; CSe+: Cesarean born, secretor. p-values were adjusted for multiple comparisons using the Benjamini and Hochberg method

^a^ adjusted for age at inclusion using ANCOVA (analysis of covariance)

^b^ adjusted for age at inclusion using a non-parametric ANCOVA (ranked analysis of covariance or Quade test)

*p < 0.05.

**Fig 3:**
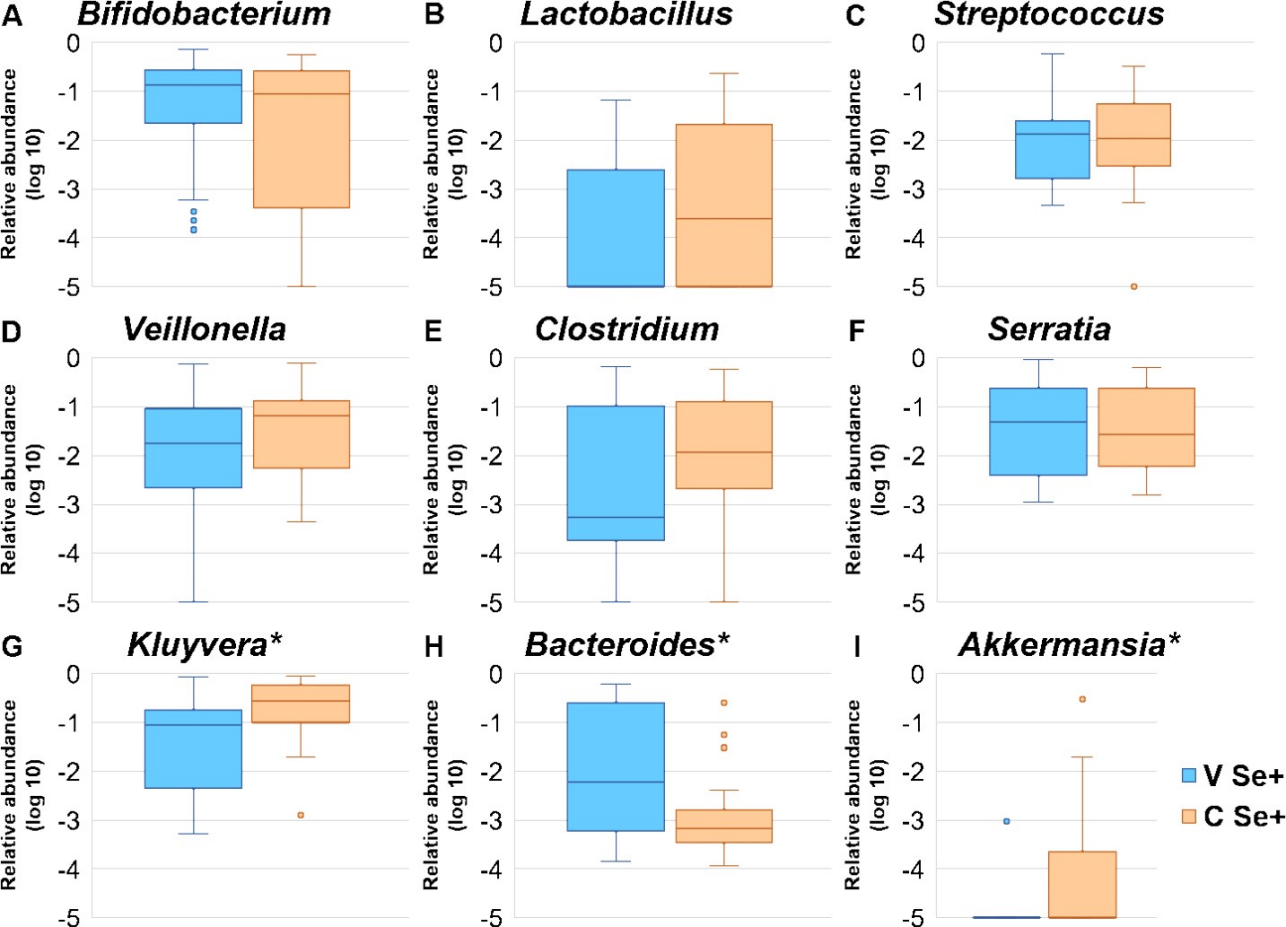
The average relative abundance of the most abundant bacterial genera in infants from secretor mothers, by birth mode. VSe+: Vaginally born (blue); CSe+: Cesarean born (yellow). The asterisks indicate statistical significance at *p* < 0.05.

Bacteroidetes and its main genus *Bacteroides* (Fig 3H) were significantly lower, whereas Verrucomicrobia and its main genus *Akkermansia* (Fig 3I) were significantly higher in CSe+ infants. The prevalence of Verrucomicrobia was significantly higher in CSe+ infants when compared to the VSe+ (29.6% and 4.8%, respectively, Fisher’s exact test, *p* < 0.001). A high abundance of Proteobacteria was observed in both CSe+ (> 40%) and VSe+ (> 30%), of which *Serratia* and *Kluyvera* were the most abundant genera. While no differences were observed in *Serratia* abundance between the groups, CSe+ infants presented a significantly higher *Kluyvera* abundance (Table 3, Fig 3G). There were no significant differences in the average relative abundance of other bacterial genera between CSe+ and VSe+ (Fig 3A–3F).

In addition to the relative abundances of the main phyla and genera, there were no differences in the absolute amounts measured by qPCR of six *Bifidobacterium* species between CSe+ and VSe+, except for *Bifidobacterium longum* (Table 4). However, CSe+ group presented a lower (but not significant) prevalence of *B*. *longum* than VSe+ (22.2% vs. 52.4%, respectively; *p* = 0.062), and differences in absolute amounts between CSe+ and VSe+ were no longer significant when considering only the samples that presented a quantifiable amount of *B*. *longum* (6.00×10^5^ cells/g *vs*. 6.66 ×10^5^ cells/g, respectively; *p* = 0.725).

**Table 4:** Prevalence and absolute amounts of selected species obtained by qPCR from the feces of vaginally and cesarean born, exclusively breastfed infants from secretor mothers.

Species	Prevalence, *n* (%)			Absolute amounts, cells/g (median (p25 –p75)) ^k^		
	VSe+ (*n* = 21)	CSe+ (*n* = 27)	*p*	VSe+ (*n* = 21)	CSe+ (*n* = 27)	*p* ^l^
*Bifidobacterium infantis*	11 (52.4)	13 (48.15)	1.000 ^i^	4.00 ×10^4^ (0–1.55 ×10^5^)	0 (0–1.08 ×10^5^)	0.950
*Bifidobacterium longum*	11 (52.4)	6 (22.2)	0.062 ^i^	2.94 ×10^4^ (0–8.67 ×10^5^)	0 (0–0)	0.009*
*Bifidobacterium bifidum* ^a^	20 (100.0)	21 (100.0)	NA	7.76 ×10^7^ (4.51 ×10^6^–3.34 ×10^8^)	3.65 ×10^7^ (4.71 ×10^5^–1.56 ×10^8^)	0.708
*Bifidobacterium breve* ^*b*^	3 (14.3)	6 (23.1)	0.711 ^j^	0 (0–0)	0 (0–8.70 ×10^2^)	0.342
*Bifidobacterium catenulatum*^c^	1 (4.8)	1 (4.0)	1.000^j^	0 (0–0)	0 (0–0)	0.903
*Bifidobacterium adolescentis*^d^	12 (60.0)	14 (58.3)	0.845 ^j^	2.70 ×10^4^ (0–1.73 ×10^7^)	2.58 ×10^4^ (0–2.39 ×10^6^)	0.769
*Bacteroides fragilis* ^*e*^	7 (35.0)	0 (0.0)	0.003* ^j^	0 (0–2.48 ×10^6^)	NA	NA
*Clostridioides difficile* ^f^	5 (25.0)	9 (45.0)	0.320 ^j^	0 (0–3.84 ×10^4^)	0 (0–1.16 ×10^4^)	0.229
*Escherichia coli* ^*g*^	16 (80.0)	14 (66.7)	0.541 ^j^	1.86 ×10^6^ (4.12 ×103–1.79 ×10^7^)	5.01 ×10^4^ (0–5.15 ×10^6^)	0.820
*Methanobrevibacter smithii* ^*h*^	6 (30.0)	14 (61.0)	0.086 ^j^	0 (0–1.04 ×10^4^)	2.30 × 10^3^ (0–3.39 ×10^4^)	0.071

VSe+: Vaginally born, secretor; CSe+: Cesarean born, secretor; NA: Not applicable

^a^ Missing data from 1 infant from VSe+ group and 6 infants from CSe+ group

^b^ Missing data from 1 infant from group CSe+

^c^ Missing data from 2 infants from group CSe+

^d^ Missing data from 1 infant from VSe+ group and 3 infants from CSe+ group

^e^ Missing data from 1 infant from VSe+ group and 5 infants from CSe+ group

^f^ Missing data from 1 infant from VSe+ group and 7 infants from CSe+ group

^g^ Missing data from 1 infant from VSe+ group and 6 infants from CSe+ group

^h^ Missing data from 1 infant from VSe+ group and 4 infants from CSe+ group

^i^ Chi-square test

^j^ Fisher exact test

^k^ Zero/non-detected levels were included as zero values in the analysis. The detection limit was 1 cell/g for all organisms

^l^ p-values were adjusted for multiple comparisons using the Benjamini and Hochberg method and for age at inclusion using a non-parametric ANCOVA (ranked analysis of covariance or Quade test)

*p<0.05.

There were no significant differences in the prevalence and amounts of other bacterial species of clinical relevance such as *Escherichia coli*, *Clostridioides difficile* and *Methanobrevibacter smithii* (Table 4). The prevalence of *Bacteroides fragilis* was significantly lower in CSe+ than VSe+ infants (0.0% *vs*. 35.0%, respectively; *p* = 0.003), since it was not detected in any fecal sample from CSe+ infants, corroborating the 16S sequencing results.

## Discussion

In this study, we compared the fecal microbiota of 48 full-term, healthy, exclusively breastfed infants from secretor mothers, according to the mode of birth. Cesarean born infants presented lower abundances of *Bacteroides*, lower prevalence of *B*. *longum* and higher abundances of *Akkermansia and Kluyvera*. The overall microbiota composition (alpha and beta diversity) was not different between cesarean and vaginally born infants breastfed by secretor mothers.

On the contrary to previous studies comparing the fecal microbiota of vaginally and cesarean born infants without considering the maternal secretor status [6,7], we did not observe differences in *Bifidobacterium* relative abundances between CSe+ and VSe+ infants. Besides the relative amounts obtained by 16S sequencing, we measured absolute numbers of some clinically relevant groups through qPCR, including six *Bifidobacterium* species, of which a difference between CSe+ and VSe+ infants was observed only in the concentration of *B*. *longum*. Previous studies reported that the presence of α1–2 fucosylated HMOs in breast milk (or maternal Se+ phenotype) affects *Bifidobacterium* establishment and abundance in the infant’s gut, being higher in secretor-fed infants [22,23]. This may explain the lack of difference in the relative abundances and counts of *Bifidobacterium* between CSe+ and VSe+ in our study. *Bifidobacterium* is essential for inhibiting the growth of pathogenic organisms, modulating mucosal barrier function, and promoting immunological and inflammatory responses in the infant’s gut [33,34]. The perturbance of *Bifidobacterium* establishment during the neonatal period may be involved in the development of immune diseases, such as eczema [35]. *Bifidobacterium* is also a component of the human milk microbiota [36], and its occurrence in the fecal microbiota of CSe+ infants observed in our study suggests that human milk may be an important source of *Bifidobacterium* besides the birth canal, contributing to the establishment of this genus in the infant gut microbiota.

Our results broadly support the findings of the recent and only previous published study, conducted in Finland, about the effects of maternal secretor phenotype on the microbiota of cesarean and vaginally born infants [13]. The authors reported that cesarean born infants from Se+ mothers presented a more modest deviation in the overall microbiota composition, compared to those of non-secretor mothers. Similar to our findings, the authors reported that CSe+ had significantly lower abundances of Bacteroidetes and *Bacteroides* and significantly higher abundances of Verrucomicrobia and *Akkermansia muciniphila* in their gut microbiota, compared to VSe+ infants. Considering that ethnicity and environmental factors are strongly associated with the microbial composition and function in healthy individuals [37], it is remarkable that similar associations were observed in such distinct populations as the Nordic and Brazilian. Differently, they found significantly lower Actinobacteria and *Bifidobacterium* and higher Firmicutes abundances in CSe+ compared to VSe+, which were not observed in our cohort [13].

The significantly higher abundance of Verrucomicrobia and its main genus *Akkermansia* observed only in CSe+ infants is a curious finding of both studies. *Akkermansia* is a mucin-degrading bacterium present in the human intestinal tract, which is involved in immune regulation and promotes the gut barrier function [38]. Lower abundance and prevalence of *Akkermansia* have been associated with obesity and related metabolic complications [39] and also with allergic diseases in children, such as atopy and asthma [40,41]. *Akkermansia* is also a component of the breast milk microbiota, and positive correlations between α1–2 fucosylated HMOs and *Akkermansia muciniphila* in human colostrum have been reported, as well as the ability of *Akkermansia* to degrade HMOs [42]. Interestingly, a study showed that CSe+ infants with high hereditary allergy risk, but who did not develop IgE allergic disease and IgE eczema at two years, consumed breast milk with higher levels of 2’-FL, suggesting a protective role of α1–2 fucosylated HMOs in the manifestation of atopy [43]. A possible explanation for this potential lower allergy risk in CSe+ infants is the higher abundance of *Bifidobacterium* and *Akkermansia* in the microbiota of CSe+ infants demonstrated in our study.

Some studies have reported impairment on *Bacteroides* transmission from mother to child by cesarean section [6–8,44], which remains absent from the microbiota at 12 months of age [7]. Interestingly, we also observed a significantly lower abundance of *Bacteroides* and the absence of *B*. *fragilis* in CSe+ compared to VSe+ infants, indicating that even exclusive breastfeeding and the α1–2 fucosylated HMOs cannot repair the disruption on *Bacteroides* transmission from mother to child caused by cesarean section. Cesarean birth has been associated with neurodevelopmental and psychiatric disorders, such as autism spectrum disorder and attention-deficit/hyperactivity disorder [45]. Curiously, *Bacteroides* is a producer of the neurotransmitter γ-aminobutyric acid (GABA)–whose misregulation has been linked to mental diseases–and low fecal *Bacteroides* levels were associated with brain signatures of depression [46]. In an experimental model, oral treatment with *Bacteroides fragilis* attenuated the behavioral symptoms of autism [47]. Besides neurological effects, an association among cesarean birth, delayed Bacteroidetes colonization, and reduced Th1 responses have also been described [44]. Further studies are needed to investigate the clinical consequences of *Bacteroides* deprivation at the neonatal period and early infancy.

We observed a high abundance of Proteobacteria in our cohort, of which *Serratia* and *Kluyvera* were the main genera. While Proteobacteria is one of the main phyla commonly reported in infant fecal samples [7,13], high abundances of *Serratia* and *Kluyvera* have not been reported in the literature. *Serratia* has been described as one of the dominant bacterial genera in human milk [48] and was reported to be highly abundant in breast milk from women with mastitis [49]. *Kluyvera* has been reported as a cause of pediatric infections [50,51]. Previous studies conducted in Brazil found a predominance of *Escherichia* in the fecal microbiota of healthy, vaginally born, one-month-old exclusively breastfed infants from low socioeconomic levels [52,53]. Besides *Escherichia*, the authors did not observe significant amounts of other members of the Proteobacteria phylum [52]. This difference to our study may be attributed to the middle-high socioeconomic level of our cohort, which possibly determines a different environmental exposure since the infants from these studies were also exclusively breastfed. However, the relative abundances of other principal members of the infant microbiota reported by these previous studies were very similar to the VSe+ group of our research, especially *Bacteroides* (~10%), *Veillonella* (~10%), and *Streptococcus* (~5%) [52].

Another novel finding of our study is the occurrence of *Methanobrevibacter smithii* on the microbiota of exclusively breastfed infants, independent of the birth mode. In previous studies, our group reported higher concentrations of *M*. *smithii* in the feces of school-aged children living near a landfill, from families of low socioeconomic level and poor sanitary housing conditions [54,55]. In the present study, we demonstrate that *M*. *smithii* is already present in the neonatal period in exclusively breastfed infants, but in lower levels than in school-aged children.

The limitations of the study include the absence of a bead-beating step during the DNA extraction, which may lead to an under-detection of *Bifidobacterium*. However, *Bifidobacterium* was still detected in high abundances, composing one of the main bacterial genera, both in VSe+ and CSe+ infants. The low prevalence of the non-secretor phenotype required a larger sample to compare the four groups according to the birth mode and maternal secretor status. Because of the cross-sectional design of the study, we were unable to ascertain whether the partial microbiota recovery in CSe+ infants is sustained or if other changes occur over time. Although the exposure to antibiotics was an exclusion criterion for the infants, we did not collect data on the maternal use of intra and postpartum antibiotics, which can be considered a limitation. Among the strengths of our study is our cohort, composed of healthy and exclusively breastfed infants, avoiding the bias on microbiota composition introduced by mixed feeding. Other strengths include the identification of the maternal secretor phenotype through the fucosylated HMOs profile and the use of high-throughput genetic sequencing complemented with the absolute quantification of the main bacterial genera and species by qPCR to profile the infant gut microbiota. Besides, we obtained a more balanced distribution of cesarean and vaginally born infants relative to previous studies.

In conclusion, our results show that there is no difference in the overall gut microbiota composition (alpha and beta diversity) and *Bifidobacterium* amounts between vaginally and cesarean born infants fed with breast milk containing the α1–2 fucosylated HMOs fraction. Differences between VSe+ and CSe+ infants were still observed in the abundances of the phyla Bacteroidetes and Verrucomicrobia, in the genera *Bacteroides*, *Akkermansia*, and *Kluyvera* and the species *B*. *longum*. Further studies are necessary to investigate the potential benefits of the HMOs on the microbiota of cesarean born infants.

## Supporting information

S1 Fig Flowchart of the participants selected for the subset of the present study. CIAAM/UNIFESP, Breastfeeding Incentive and Support Center/Universidade Federal de São Paulo.
(PDF)

S1 Table Concentrations (g/L) of α1–2 fucosylated HMOs in breast milk according to the mode of birth and maternal secretor status. Values are presented as median (p25 –p75) unless otherwise indicated; * statistical comparisons between VSe+ and CSe+; 2’-FL: 2’-fucosyllactose; LNFP I: lacto-N-fucopentaose I; LNDFH I: lacto-N-difucohexaose I; DFLNHc: difucosyllacto-N-hexaose c; ^a^ breast milk from these groups presented levels of α1–2 fucosylated HMOs below the quantification limit; ^b^ quantification limit: 0.000039 g/L; ^c^ quantification limit: 0.000156 g/L; ^d^ Mann-Whitney rank sum test; ^e^ Student’s t test.
(PDF)

S2 Table Primers used for the qPCR quantification of the main genera and species obtained from the infant fecal microbiota. (PDF)
