# Supplementary figures and images for: Gut microbiota comparison of vaginally and cesarean born infants exclusively breastfed by mothers secreting α1–2 fucosylated oligosaccharides in breast milk
Source: PLoS One. 2021 Feb 8;16(2):e0246839. doi: 10.1371/journal.pone.0246839 (PMC7870049; doi:10.1371/journal.pone.0246839)

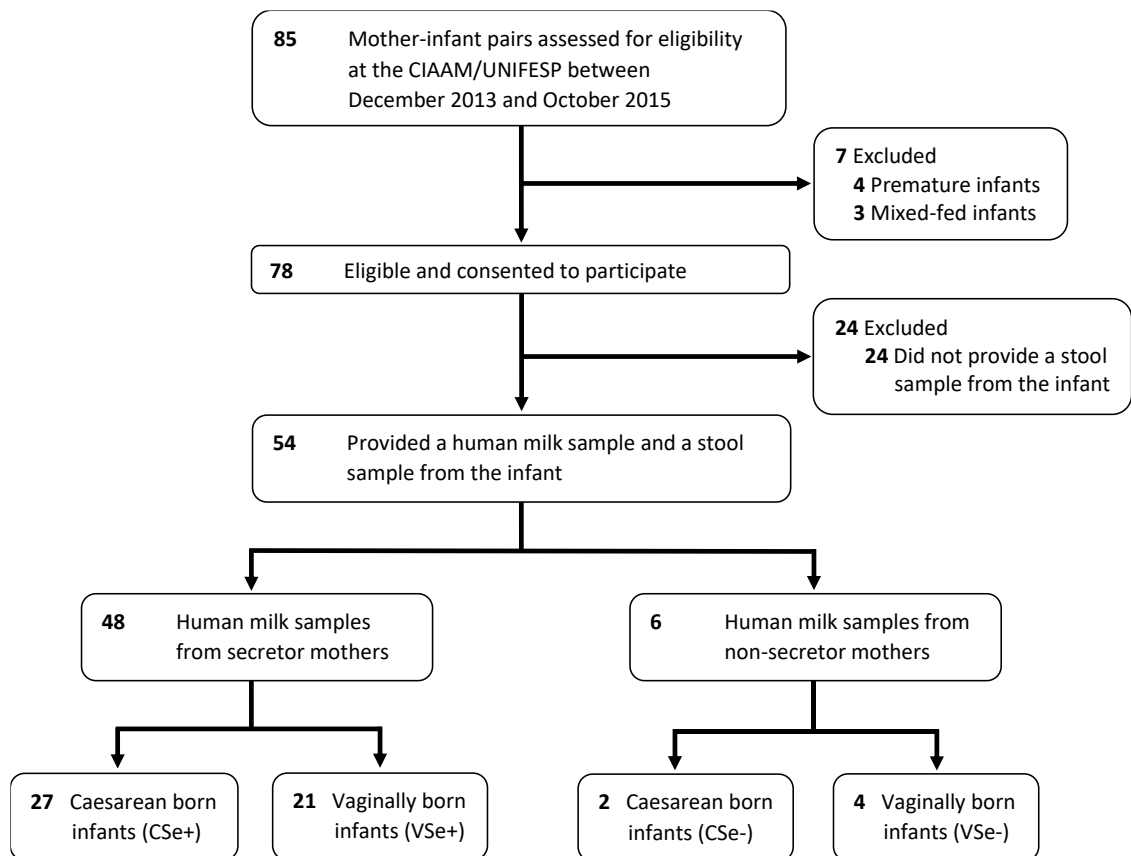

Supplement: S1 Fig — CIAAM/UNIFESP, Breastfeeding Incentive and Support Center/Universidade Federal de São Paulo. (PDF) [file pone.0246839.s001.pdf]
