# Supplementary material for: Gut microbiota comparison of vaginally and cesarean born infants exclusively breastfed by mothers secreting α1–2 fucosylated oligosaccharides in breast milk
Source: PLoS One. 2021 Feb 8;16(2):e0246839. doi: 10.1371/journal.pone.0246839 (PMC7870049; doi:10.1371/journal.pone.0246839)
Supplement: S1 Table — Values are presented as median (p25 –p75) unless otherwise indicated; * statistical comparisons between VSe+ and CSe+; 2’-FL: 2’-fucosyllactose; LNFP I: lacto-N-fucopentaose I; LNDFH I: lacto-N-difucohexaose I; DFLNHc: difucosyllacto-N-hexaose c; a breast milk from these groups presented levels of α1–2 fucosylated HMOs below the quantification limit; b quantification limit: 0.000039 g/L; c quantification limit: 0.000156 g/L; d Mann-Whitney rank sum test; e Student’s t test. (PDF) [file pone.0246839.s002.pdf]

α1-2 fucosylated HMOs	VSe+ (*n* = 21)	CSe+ (*n* = 27)	VSe- (*n* = 4) [a]	CSe- (*n* = 2) [a]	*p**
2'-FL [b]	2.653 (1.677 – 3.754)	2.127 (1.472 – 2.572)	–	–	0.085[d]
LNFP I [c]	0.736 (0.444 – 1.564)	0.511 (0.269 – 0.962)	–	–	0.025[d]
LNDFH I (mean (SD))[b]	0.823 (0.527)	0.986 (0.627)	–	–	0.342[e]
DFLNHc [b]	0.0614 (0.0119 – 0.0846)	0.0577 (0.0226 – 0.0931)	–	–	0.851[d]
