# Supplementary material for: Gut microbiota comparison of vaginally and cesarean born infants exclusively breastfed by mothers secreting α1–2 fucosylated oligosaccharides in breast milk
Source: PLoS One. 2021 Feb 8;16(2):e0246839. doi: 10.1371/journal.pone.0246839 (PMC7870049; doi:10.1371/journal.pone.0246839)
Supplement: S2 Table — (PDF) [file pone.0246839.s003.pdf]

Genera/species	Sequences (5' – 3')	Reference
B. bifidum	F: CTCCTGGAAACGGGTGG	[1]
	R: GGTGTTCTTCCCGATATCTACA	
B. infantis	F: CGCGAGCAAAACAATGGTT	[2]
	R: AACGATCGAAACGAACAATAGAGTT	
B. longum	F: TGGAAGACGTCGTTGGCTTT	[2]
	R: ATCGCGCCAGGCAAAA	
B. breve	F: GTGGTGGCTTGAGAACTGGATAG	[2]
	R: CAAAACGATCGAAACAAACACTAAA	
B. catenulatum	F: GTGGACGCGAGCAATGC	[2]
	R: AATAGAGCCTGGCGAAATCG	
B. adolescentis	F: ATAGTGGACGCGAGCAAGAGA	[2]
	R: TTGAAGAGTTTGGCGAAATCG	
Bacteroides fragilis	F: ATAGCCTTTCGAAAGRAAGAT	[3]
	R: CCAGTATCAACTGCAATTTTA	
Clostridium difficile	F: TTGAGCGATTTACTTCGGTAAAGA	[1]
	R: CCATTCTGTACTGGCTCACCT	
Escherichia coli	F: CATGCCGCGTGTATGAAGAA	[4]
	R: CGGGTAACGTCAATGAGCAAA	
Methanobrevibacter	F: GAAAGCGGAGGTCCTGAA	[5]
smithii	R: ACTGAAAAACCTCCGCAAAC	

References

1. Schäffler H, Kaschitzki A, Alberts C, Bodammer P, Bannert K, Köller T, et al. Alterations in the mucosa-associated bacterial composition in Crohn's disease: a pilot study. Int J Colorectal Dis [Internet]. 2016;31:961–71. Available from: http://link.springer.com/10.1007/s00384-016-2548-z

2. Haarman M, Knol J. Quantitative real-time PCR assays to identify and quantify fecal Bifidobacterium species in infants receiving a prebiotic infant formula. Appl Environ Microbiol [Internet]. 2005 [cited 2013 Jul 31];71:2318–24. Available from: http://aem.asm.org/content/71/5/2318.short

3. Matsuki T, Watanabe K, Fujimoto J, Miyamoto Y, Takada T, Matsumoto K, et al. Development of 16S rRNA-gene-targeted group-specific primers for the detection and identification of predominant bacteria in human feces. Appl Environ Microbiol [Internet]. 2002 [cited 2013 Aug 1];68:5445–51. Available from:

http://aem.asm.org/content/68/11/5445.short

4. Huijsdens XW, Linskens RK, Mak M, Meuwissen SGM, Vandenbroucke-Grauls CMJE, Savelkoul PHM. Quantification of bacteria adherent to gastrointestinal mucosa by real-time PCR. J Clin Microbiol [Internet]. 2002;40:4423–7. Available from: http://www.ncbi.nlm.nih.gov/pubmed/12454130

5. Johnston C, Ufnar JA, Griffith JF, Gooch JA, Stewart JR. A real-time qPCR assay for the detection of the nifH gene of Methanobrevibacter smithii, a potential indicator of sewage pollution. J Appl Microbiol [Internet]. 2010;109:1946–56. Available from: http://www.ncbi.nlm.nih.gov/pubmed/21070516
